# Supplementary material for: Region 4 of the RNA polymerase σ subunit counteracts pausing during initial transcription
Source: J Biol Chem. 2021 Jan 8;296:100253. doi: 10.1074/jbc.RA120.016299 (PMC7948647; doi:10.1074/jbc.RA120.016299)
Supplement: Supplemental Figures S1–S6 [file mmc1.pdf]

Region 4 of the RNA polymerase σ subunit counteracts pausing during initial transcription

Konstantin Brodolin, Zakia Morichaud

IRIM, CNRS, Univ Montpellier, 1919 route de Mende 34293 Montpellier, France

Supplementary figures and legends

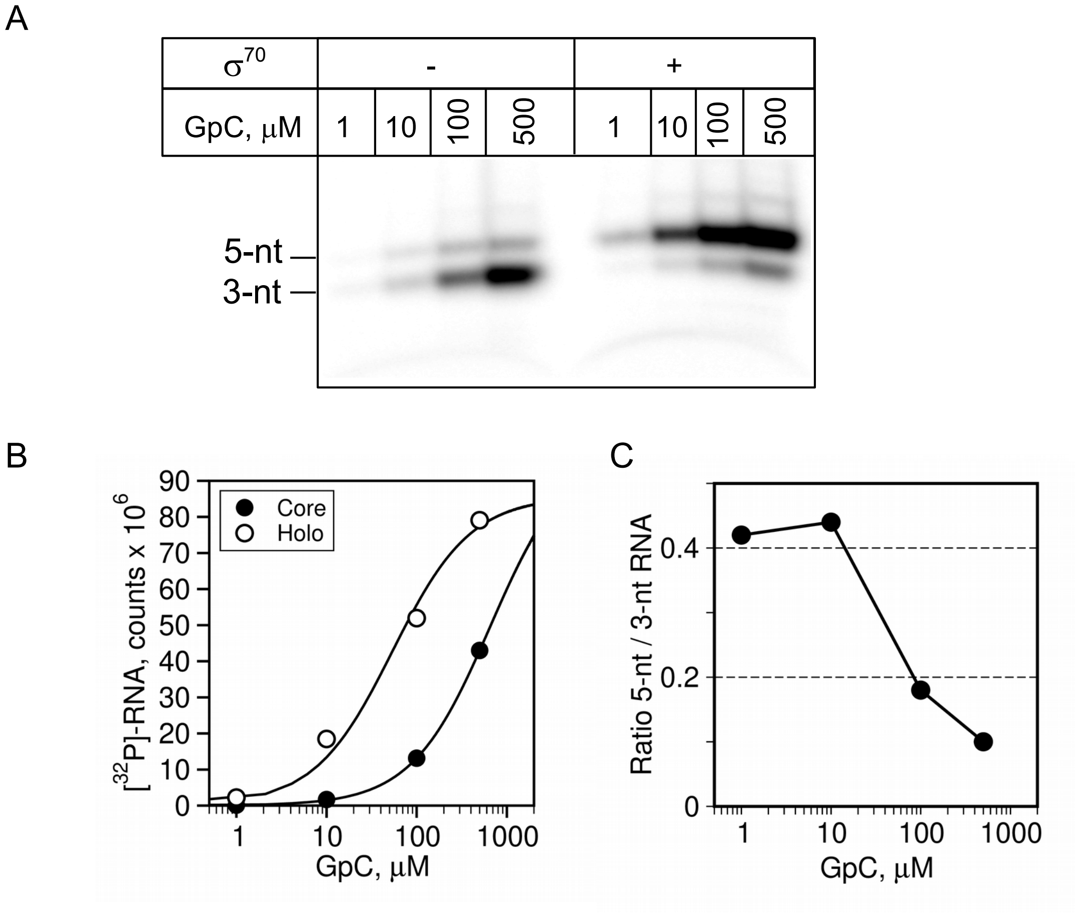

Figure S1. Effect of the pRNA concentration on translocation efficiency.
(A). Primer extension by the *Eco*RNAP core and σ^{70}-*Eco*RNAP holoenzyme performed at the SDT scaffold with indicated concentrations of pRNA (GpC) and fixed concentration of [α^{32}P]-UTP, GTP and ATP. Transcription was performed for 2 min. (B) Quantification of the experiment shown in panel A. Cumulative RNA products are plotted as function of pRNA concentration. (C). Ratio of the 5-nt to 3-nt RNAs for reactions with *Eco*RNAP core plotted as function of pRNA concentration.

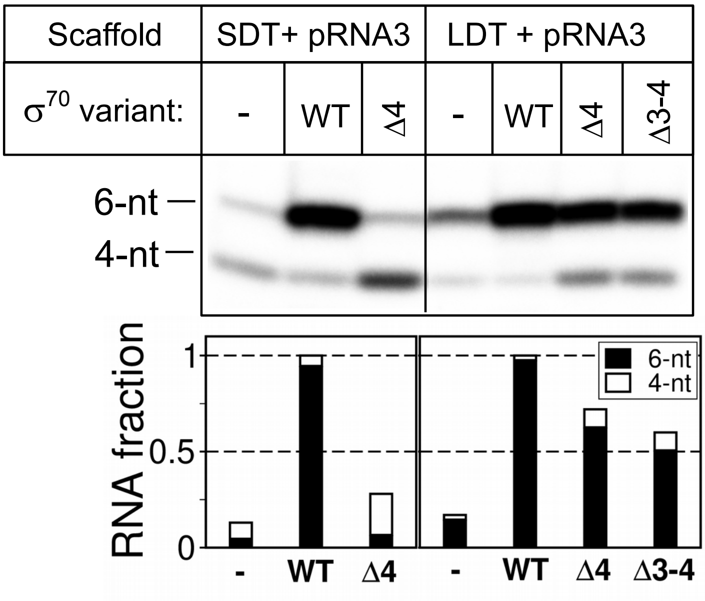

Figure S2. The dwDNA duplex promotes forward translocation.

Extension of 3-nt pRNA (pRNA3) by *Eco*RNAP on the SDT and LDT scaffolds in presence of the mutant σ^{70} variants. Stacked bar graph shows quantification of the RNA products. Values of each RNA product were normalized to the total [^{32}P]-RNA synthesized in the presence of the full length σ^{70} subunit.

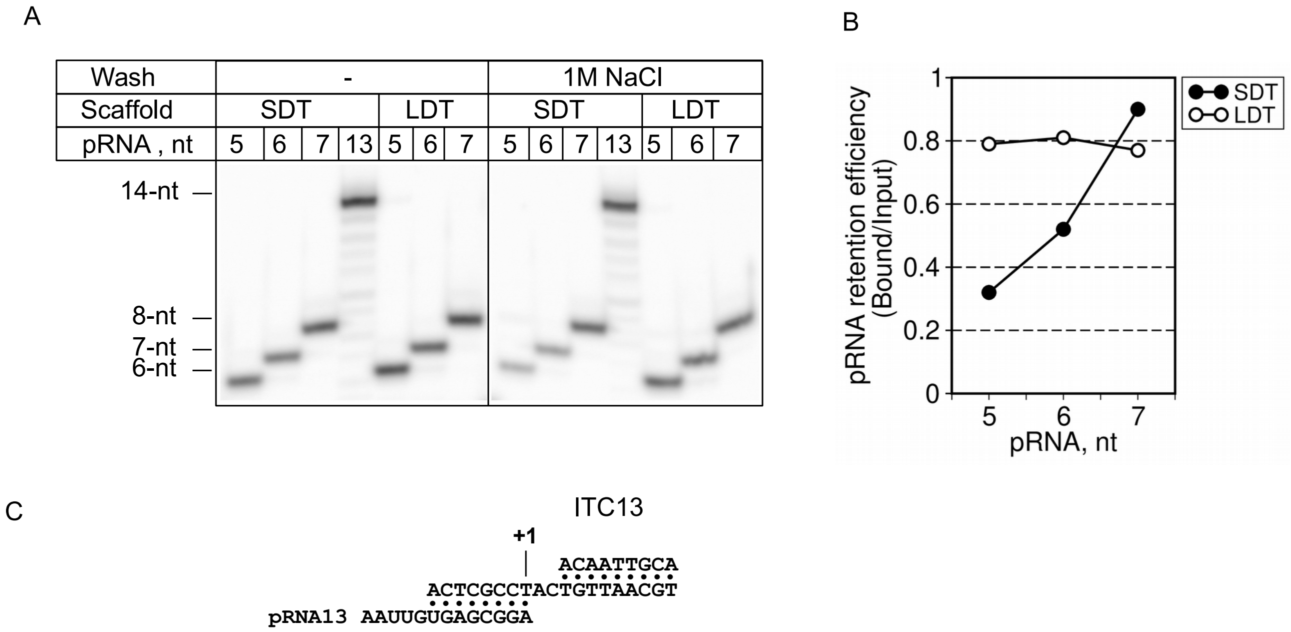

Figure S3. RNA retention in ITCs formed by the *Eco*RNAP core.

(A). [α^{32}P]-UTP labeled RNAs produced in primer extension reactions in ITC5, ITC6, ITC7 (5- 7-nt pRNA shown in **Fig. S4A**) and ITC13 (shown in panel C) assembled on SDT and LDT scaffolds. ITCs were immobilized on Ni^{2+}-agarose beads and washed with buffer containing 1M NaCl. (B). The fraction of labeled RNA retained in ITCs in panel A was calculated as a ratio between RNA in the washed ITCn to the RNA in the unwashed ITCn. (C). Scheme of the SDT scaffold with 13-nt pRNA used to assemble ITC13.

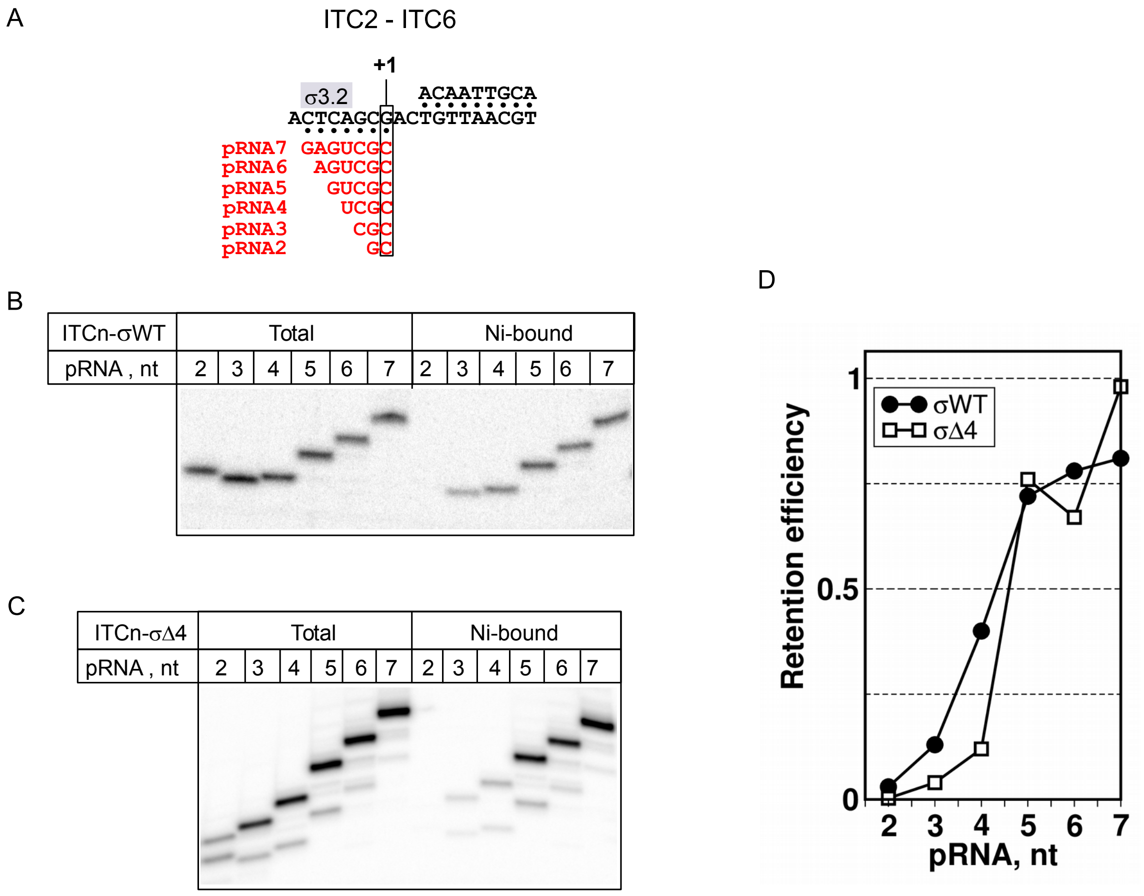

Figure S4. The σ subunit region 4 stabilizes short RNAs in ITCs formed on SDT scaffold.

(A). Scheme of the SDT scaffold with RNA primers used to assemble ITC2 - ITC7. (B and C). [α^{32}P]-UTP labeled RNAs produced in primer extension reactions in the presence of the σ^{70} (EσWT) and σΔ4 (EσΔ4) respectively. ITCs were immobilized on Ni^{2+}-agarose beads and washed with buffer, when indicated, before labeling. For the EσΔ4, GTP and ATP were added to the reactions (E). Quantification of the panels B and C. The fraction of labeled RNA retained in ITCn was calculated as a ratio between RNA in the washed ITCn to the RNA in the unwashed ITCn.

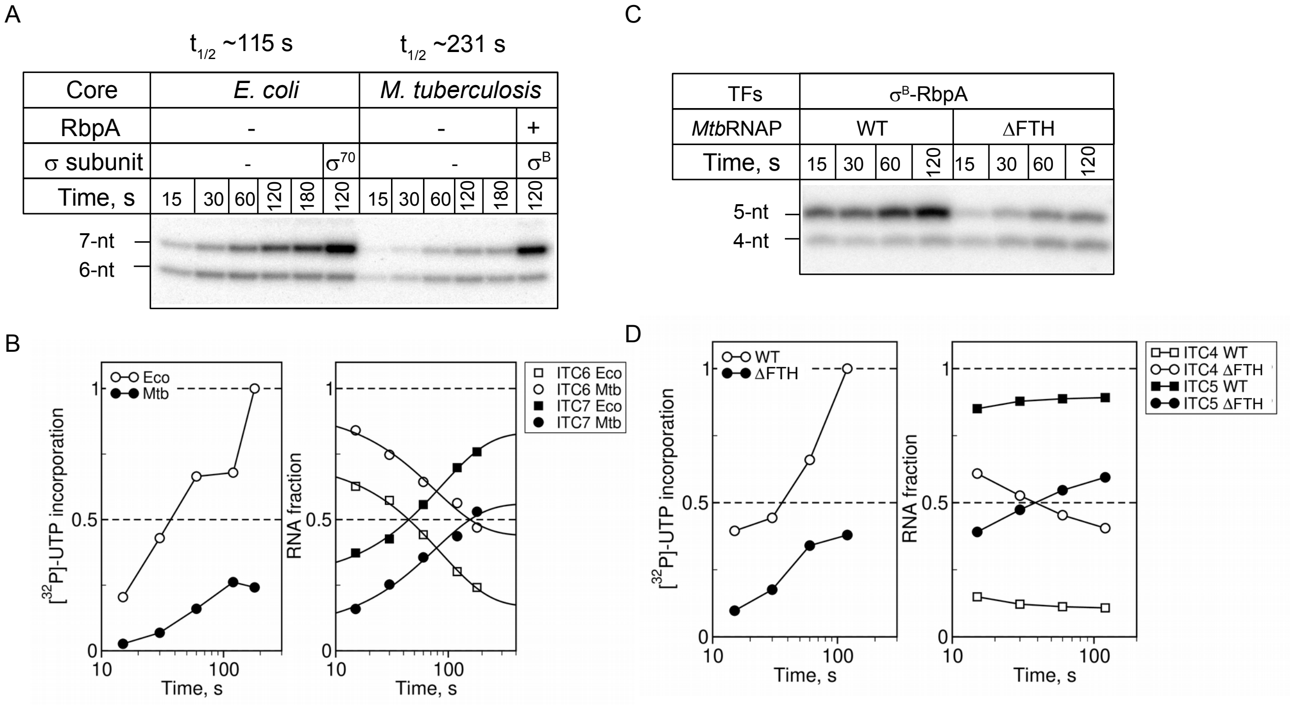

Figure S5. σ^B and β-flap modulate the initial pause.

(A). Kinetics of the 5-nt pRNA extension by the RNAP core from *E. coli* and *M. tuberculosis* at the SDT2 scaffold. Transcription was initiated by [α^{32}P]-UTP and GTP. **(B).** Quantification of the experiment shown in panel A. Left graph shows synthesis of total [^{32}P]-RNA as function of time. Right graph shows fractions of [^{32}P]-RNA products, representing various ITCs, as function of time. For each time point, values were normalized to the total [^{32}P]-RNA synthesized at that time point. (C). Kinetics of the 3-nt pRNA extension by the wild type σ^B-*Mtb*RNAP (WT) and mutant σ^B-*Mtb*RNAP$^{\Delta FTH}$ (ΔFTH) holoenzyme at SDT2 scaffold in the presence of RbpA. Transcription was initiated by [α^{32}P]-UTP and GTP. (D). Quantification of the experiment shown in panel A the same way as in panel B.

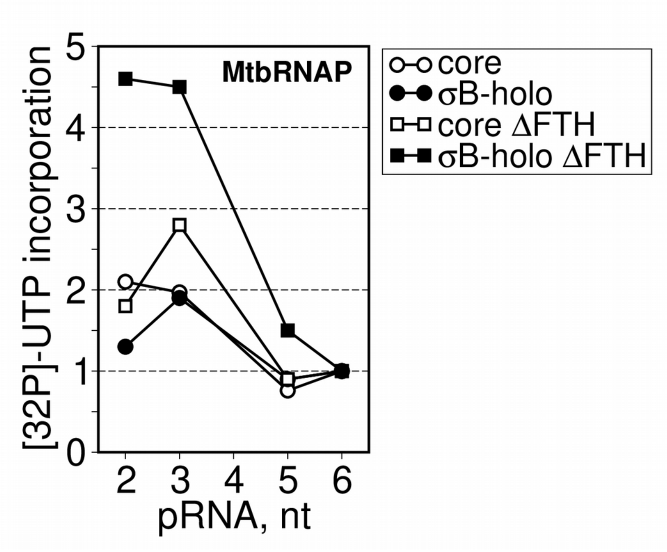

Figure S6. The σ^B subunit increases abortive transcription with the mutant *Mtb*RNAP$^{\Delta FTH}$.

Quantification of the RNA products shown in Fig. 6F. For each condition, RNA in each lane was normalized to RNA synthesized in ITC7.
